# A randomized clinical study to compare intrapleural infusion with intravenous infusion of bevacizumab in the management of malignant pleural effusion in patients with non‐small‐cell lung cancer

**DOI:** 10.1111/1759-7714.13238

**Published:** 2019-11-14

**Authors:** Keke Nie, Zhen Zhang, Yunhong You, Xingjun Zhuang, Chunling Zhang, Youxin Ji

**Affiliations:** ^1^ Department of Oncology Qingdao Cancer Hospital Qingdao China; ^2^ Department of Oncology PLA 971 Hospital Qingdao China; ^3^ Department of Oncology Qingdao Central Hospital, the Affiliated Qingdao Central Hospital of Qingdao University Qingdao China

**Keywords:** Bevacizumab, malignant pleural effusion, non‐small‐cell lung cancer, vascular endothelial growth factor

## Abstract

**Background:**

To compare the efficiency and toxicity of bevacizumab by intrapleural or intravenous infusion in the management of malignant pleural effusion in patients with non‐small‐cell lung cancer (NSCLC).

**Methods:**

Sensitizing mutation negative NSCLC patients with malignant pleural effusion were randomized into two groups in 1:1 ratio. The pleural effusion was completely drained in 24 hours; one group received intrapleural infusion and the second group received intravenous infusion of bevacizumab at a dose of 7.5 mg per kg bodyweight. The serum vascular endothelial growth factor (VEGF) was tested before and 72 hours after injection of bevacizumab. Computerized tomography (CT) scan to evaluate pleural effusions was carried out at four weeks for each patient and their survival followed‐up.

**Results:**

A total of 67 patients were screened and 43 enrolled into the study. The response rate was 80% (16 of 20) in the intrapleural group and 66.7% (14 of 21) in the intravenous group. The median duration of response (DoR) of pleural effusion was 4.50 months and 3.70 months, respectively. The median serum VEGF level at 72 hours decreased 67.25% in the intrapleural group and 57.19% in the intravenous group compared to baseline level (*P* = 0.276). The median serum VEGF level at 72 hours decreased 52.02% compared to baseline level in patients’ DoR less than three months and 68.33% in patients' DoR longer than three months, respectively (*P* = 0.014). The main side effects noted were mild to moderate hypertension, proteinuria and epistaxis.

**Conclusions:**

Bevacizumab intrapleural infusion had higher efficiency and higher safety than intravenous infusion in the management of malignant pleural effusion caused by NSCLC. The decreased level of serum VEGF at 72 hours after bevacizumab treatment was closely related to the response rate and duration of the response of pleural effusion.

## Introduction

Malignant pleural effusion (MPE) is the most severe and common complication of non‐small‐cell lung cancer (NSCLC) which is present in approximately 15% of patients with NSCLC on diagnosis, and 50% of patients will eventually develop MPE.^1^, ^2^ The occurrence of MPE often indicates late‐stage carcinoma with poor prognosis. If not properly controlled, MPE is a life‐threatening complication that will severely affect the quality of life of patients.

Vascular endothelial growth factor (VEGF) is a family of endothelial growth factors, which includes VEGFA, ‐B, ‐C, −D and ‐E, and placental growth factor.^3^ VEGF is not only the most important angiogenic factor, but also a potent stimulator that increases vascular permeability and triggers endothelial cell migration.^4^ High expression levels of VEGF have been confirmed in various normal human tissues and an increased level of VEGF has been reported in the serum of patients with numerous types of cancer and in pleural effusions due to malignant diseases.^4^ NSCLC cells can produce and secrete VEGF which promotes pleural effusion formation, angiogenesis and tumor metastatic progression.^5^ MPE is associated with high serum and plasma levels of VEGF; at present, there are no biomarkers of outcome for bevacizumab treatment in patients with MPE.^1^, ^6^

Bevacizumab (Avastin, Roche) is a vascular endothelial growth factor A (VEGFA) monoclonal antibody, which inhibits tumor angiogenesis, and is widely used in colorectal cancer and lung adenocarcinoma. Bevacizumab combined with VEGFA inhibits proliferation, migration and differentiation of vascular endothelial cells directly, attenuates VEGFA dependent tumor blood vessels formation, normalizes tumor blood vessels, prompts tumor cell apoptosis and finally shrinks the tumor.^7^ It can also promote apoptosis of endothelial cells and suppress VEGF‐induced neoangiogenesis and vascular permeability.^8^ Bevacizumab has been shown to synergize with chemotherapeutic agents to block the accumulation of pleural fluid, thus making it a potential candidate for the clinical management of MPE.^8^ Several studies have reported bevacizumab intrapleural infusion had high efficiency in the management of MPE, but the difference with intravenous infusion has yet to be revealed.

Numerous studies have indicated that high VEGF serum level is associated with pleural effusion formation, but its relationship with bevacizumab treatment response and response duration are not fully understood. This study compared the efficiency and toxicities between intrapleural and intravenous infusion of bevacizumab in order to reveal the relationship between serum VEGF levels and outcomes of pleural effusion in NSCLC.

## Methods

### Study design and patient selection

This was a multi‐center open‐label two‐arm study. Patients in the study had been pathologically or cytologically‐confirmed with locally advanced or metastatic non‐squamous NSCLC with MPE. Gene panel testing was carried out and patients with sensitizing mutations (epidermal growth factor receptor, anaplastic lymphoma kinase and ROS‐1 fusion) were excluded from the study. Measurable lesions and the size of the MPE were determined by CT scan prior to bevacizumab therapy. All eligible patients were randomly assigned into two groups in a 1:1 ratio. Patients were randomized by center. Computerized randomization was done by the Center of the Affiliated Qingdao Central Hospital of Qingdao University using Microsoft Excel 2010 formula, and dispensed to researchers case by case. If patients qualified to be included in the trial and had provided their informed signed consent, the trial center of Affiliated Qingdao Central Hospital of Qingdao University was informed and randomization carried out. All patients were treated with thoracocentesis; one group received intrapleural infusion and the other group received intravenous infusion of bevacizumab at a dose of 7.5 mg/kg bodyweight. Local therapy had to be completed at least four weeks prior to the baseline scan. All patients were 18–80 years of age with Eastern Cooperative Oncology Group (ECOG) performance status ≤3 and adequate hematological, hepatic and renal function. Tumor response was evaluated by CT every two months using the Response Evaluation Criteria in Solid Tumors Committee (RECIST) version 1.1.^9^ The response rate was taken as the percentage of patients who had a complete response (CR) or partial response (PR). The disease control rate was the summed percentage of patients with CR, PR, and stable disease (SD). Survival rates of the treated patients were examined with one year follow‐up after treatment. The numbers of survivors were expressed as a percentage of population proportion in each group of patients.

All adverse events were recorded and classified by grade according to the National Cancer Institute Common Terminology Criteria for Adverse Events version 5.0.^10^ If a patient was documented to exhibit a CR or PR, a confirmation with a second scan was required after an additional four weeks. The response of each tumor was recorded as the best tumor response observed over the entire course of treatment. Overall response rate (ORR) was defined as CR + PR.

### Chest drainage and infusion of drugs

All procedures were done at the bedside using the B‐ultrasound guidance. A pigtail catheter was inserted intercostally under appropriate local anesthetic. It was attached to a standard thoracic drainage system, and suction applied to remove pleural fluid. As much of the pleural fluid was withdrawn through the catheter in 24 hours as possible. Bevacizumab 7.5 mg/kg was dissolved in 60 mL normal saline and injected into the pleural cavity after no more pleural fluid could be withdrawn through the pigtail catheter in the intrapleural group, and bevacizumab at 7.5 mg/kg dissolved in 250 mL normal saline was administered via intravenous infusion for 90 minutes in the intravenous group.

### Measurement

The primary endpoint of the study was overall response rate (ORR) which was concluded by the rate of CR plus the rate of PR. The secondary endpoints were DoR, OS and toxicities. Tumor responses were assessed using RECIST version 1.1, MPEs were assessed by CT scan according to the American Thoracic Society criteria.^11^ Evaluation of short‐term efficacy of bevacizumab was determined according to the previous study by Kobold *et al*.^12^ Complete remission (CR): accumulated effusion had disappeared and remained stable for at least four weeks; partial remission (PR): accumulated effusion had decreased by 50% associated with improved symptoms with no increased accumulation of fluid, and remained stable for at least four weeks; remission not obvious (NC): less than 50% of the pleural effusion had disappeared, or there was no noticeable change in symptoms; progressive disease (PD): the amount of accumulated effusion had increased with worsening of symptoms. Total efficacy was calculated by the sum of CR and PR. The quality of life (QoL) was assessed by KPS and recorded as apparently improved (increase in KPS by ≥ 20 post‐treatment), improved (increase in KPS≥10), stable (no apparent change in KPS score) and reduced (KPS decline of ≥10). DoR was calculated from the time of patients with either a CR or a PR of pleural effusion to the time of progression.

Patients with no response were not included in this calculation. The size of the pleural effusion was defined as follows: Massive ‐ effusion volume >75% of the hemithorax; large ‐ effusion volume 50%–75% of the hemithorax; moderate ‐ effusion volume 25%–50% of the hemithorax; and small ‐ effusion volume <25% of the hemithorax.

Tumor measurements with CT scan for lung and abdomen or ultrasound for subcutaneous lesions and lymph nodes were performed at screening and every four weeks thereafter. Ultrasound was performed every four weeks and CT scans every two months, but it was not limited to patients whose disease was suspected to be rapidly progressing. Patients' compliance, treatment safety and side effects were assessed at each check point every four weeks.

All adverse events were recorded and classified by grade according to the National Cancer Institute Common Terminology Criteria for Adverse Events version 5.0.^10^

### Statistical analysis

This was a noninferiority study. A sample size of 156 patients was calculated for each group and a type I error of 0.05 (one‐side), 80% power of test and assumption of a 0.5 coefficient of variability at a 1:1 sample ratio of the two groups. The anticipated dropout rate was 10% and the actual value of coefficient of variability was likely to be more than 0.5, the optimum sample size would be 196 patients per group if the hazard ratio (HR) was close to 1.15 in this study. A noninferiority would be established if the HR was less than 1.15 of the intrapleural group versus the intravenous group in the full analysis set.

Based on the Cox proportional hazards model, and taking into account the influence of gender (male or female), ECOG performance status score (1 vs. 2 vs. 3), and chemotherapy regimens, HR and 95% confidence interval (CI) were calculated in the full analysis population. DoR curves were analyzed using Sigmaplot 11 (Systat software Inc., USA) Kaplan‐Meier log‐rank test and the HR using Cox proportional hazards model in the intention‐to‐treat (ITT) patients.

The response rate, symptom reduction, and treatment‐related adverse events were assessed using a Fisher's exact test (all randomly assigned patients received at least one dose of the study drug).

This study was approved by the Ethics Committee of Affiliated Qingdao Central Hospital of Qingdao University, and performed in compliance with the provisions of Good Clinical Practice guidelines, the Declaration of Helsinki and local laws. Informed consent was obtained from all patients before enrollment into the study.

## Results

**Figure 1:**
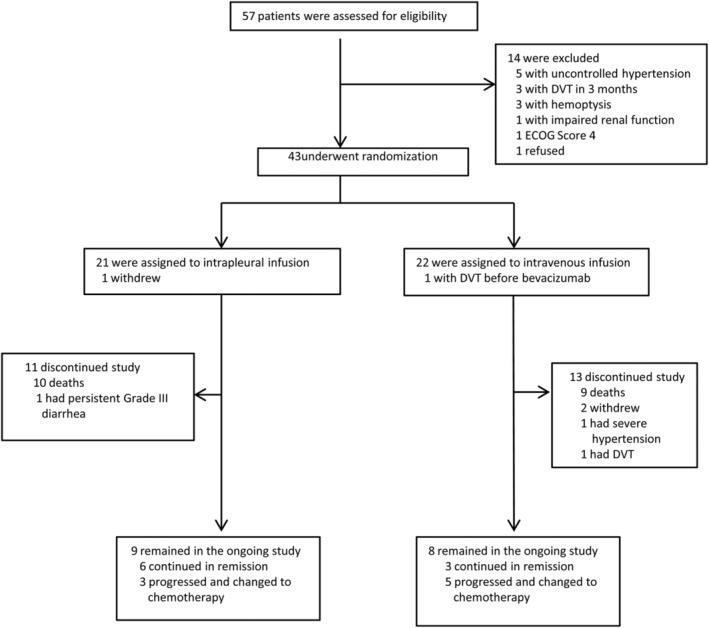
Trial profile. The cutoff date was 30 March 2019 and the last follow‐up data was obtained on 30 July 2019.

From April 2015 to March 2019, 57 patients were screened of which 43 were enrolled into the study. The remaining 14 patients were excluded during screening as they did not meet the inclusion criteria (Fig 1).

All enrolled patients were Chinese and staged as IV using the AJCC 2007 staging system. There was a total of 43 patients, of whom 27 (62.8%) were men and 16 (37.2%) women (Table 1). Study patients were pathologically or cytologically confirmed as local advanced, or metastatic nonsquamous NSCLC without acquired EGFR T790M mutation or ALK, ROS‐1 rearrangement. All patients had adequate performance status and reasonable hematologic, hepatic or renal function. The mean age of patients in this study was 62 years. Although patients’ ECOG performance status score 0–3 qualified for the study, no score 0 patient was enrolled. A reason for this may have been because enrolled patients were all in the advanced stages of disease and had large to massive pleural effusions when enrolled into the study. The last follow‐up date was 30 July 2019.

**Table 1:** Patients’ baseline characteristics

Variable	Intrapleural (*N* = 21)	Intravenous (*N* = 22)	*P‐*value
Age ‐ year	62 ± 11.0	62 ± 12.0	0.091
Sex no. (%)			0.910
Male	13 (61.9)	14 (63.6)	
Female	8 (38.1)	8 (36.4)	
Histology			0.953
Adenocarcinoma	15 (71.4)	14 (63.6)	
Large cell carcinoma	4 (19.0)	5 (22.7)	
NOS	2 (9.5)	3 (13,6)	
ECOG performance status no. (%)			0.882
1	8 (38.1)	10 (45.5)	
2	9 (42.9)	7 (31.8)	
3	4 (19.0)	5 (22.7)	
Previous treatment	13 (61.9)	13 (59.1)	0.887
Chemotherapy	10 (47.6)	9 (40.9)	
Systemic bevacizumab	5 (23.8)	5 (22.7)	
Chest irradiation	4 (19.0)	6 (27.3)	

ECOG, Eastern Cooperative Oncology Group performance status; NOS, not otherwise specified.

In this study, one patient withdrew from the intrapleural group and one patient could not be included because deep venous thrombosis (DVT) of the left lower extremity was present before bevacizumab infusion in the intravenous group. A total of 41 patients were evaluated for treatment response. A total of 15 of 41 were treatment naïve patients, 12 with ECOG score ≤ 2 were treated with cisplatin/carboplatin and pemetrexed on the day after bevacizumab infusion, and two were treated with pemetrexed only because they were over 75‐years‐old. There were 19/41 patients who had previously received platinum‐containing doublet chemotherapy, which was combined with bevacizumab in 10 patients. A total of 9/41 patients had an ECOG score of three. Three of these were treatment naïve, six had recurrence after chemotherapy who were treated by withdrawal of pleural effusion plus bevacizumab infusion only. The ORR (CR+PR) was 80.0% (16 of 20), the CR rate was 40.0% (eight of 20) in the intrapleural group; and ORR (CR+PR) was 66.7% (14 of 21) and CR rate was 19.0% (four of 21) in the intravenous group (Table 2).

**Table 2:** Summary of efficiency measures

Outcome	Intrapleural group (*N* = 20)	Intravenous group (*N* = 21)	Hazard ratio (95% CI)	*P*‐value
Serum VEGF decreased 72 hours to baseline level (%)	67.25	57.19	0.628 (0.261–0.701)	0.295
Duration of response (M ± SE)	4.54 ± 0.52	3.69 ± 0.30	3.101–4.284	0.276
Serum VEGF decreased in patients DoR ≥ 3 m versus < 3 m (%)	68.33	52.02	0.526 (0.200–1.384)	0.014
Type of response no. (%)				
ORR (CR + PR)	16 (80.0)	14 (66.7)		0.586
CR (%)	8 (40.0)	4 (19.0)		
PR (%)	8 (40.0)	10 (47.6)		
SD (%)	3 (15.0)	3 (14.3)		
DCR (CR + PR + SD) (%)	19 (95.0)	17 (81.0)		0.306

CR, complete response; DCR, disease control rate; DoR, duration of response; PR, partial response; SD, stable disease; VEGF, vascular endothelial growth factor.

**Figure 2:**
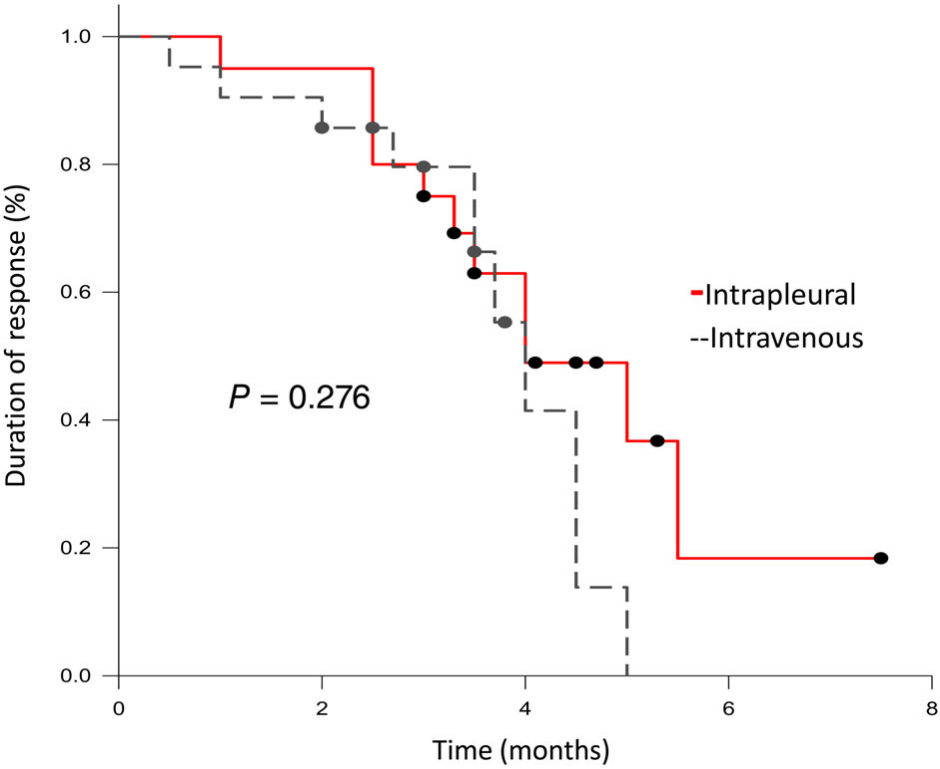
Kaplan‐Meier analysis of duration of response in the full analysis set.

Patients’ serum VEGF levels were tested on the day before bevacizumab injection as baseline and 72 hours after bevacizumab injection. Serum VEGF levels decreased significantly at 72 hours after bevacizumab injection compared to baseline levels. The median serum VEGF level decreased 67.25% in the intrapleural group and 57.19% in the intravenous group, but there was no significant difference (HR 0.628; 95% confidence interval [CI], 0.261–0.701; *P* = 0.295). The median DoR was 4.5 months (95% CI, 3.520–5.566) in the intrapleural group and 3.7 months (95% CI, 3.101–4.284) in the intravenous group, but there was no significant difference (*P* = 0.276) (Fig 2).

**Figure 3:**
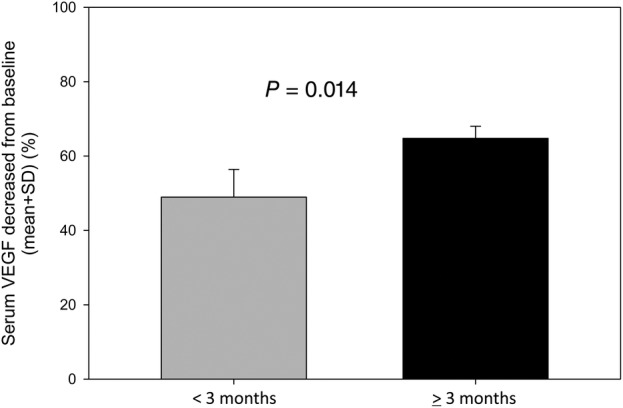
Serum VEGF decreased from baseline according to three months duration of response in the study population.

The median serum VEGF decreased level was 52.02% in patients’ DoR <3 months and 68.33% in that ≥3 months, respectively, and there was a significant difference (HR 0.526; 95% confidence interval [CI], 0.200–1.384; *P* = 0.014) (Fig 3).

The main adverse effects related to bevacizumab use were hypertension, proteinuria and epistaxis, which occurred more in the intravenous than the intrapleural group (Table 3).

**Table 3:** Summary of adverse events

	Intrapleural (*N* = 20)		Intravenous (*N* = 21)		*P*‐value	
Adverse event	All grade	Grade 3 or 4	All grade	Grade 3 or 4	Any grade	Grade 3 or 4
	*Number (percent)*					
Hoarseness	1 (5.0)	0 (0)	5 (23.8)	1 (4.8)	<0.01	N/A
Hypertension	2 (10.0)	0 (0)	6 (28.6)	3 (14.3)	<0.01	<0.01
Proteinuria	2 (10.0)	0 (0)	3 (14.3)	0 (0)	<0.01	N/A
Epistaxis	0 (0)	0 (0)	2 (9.5)	0 (0)	N/A	N/A
Anorexia	14 (70.0)	2 (10.0)	15 (71.4)	3 (14.3)	>0.05	>0.05
Nausea	13 (65.0)	3 (15.0)	14 (66.7)	3 (14.3)	>0.05	>0.05
Vomiting	3 (15.0)	1 (5.0)	5 (23.8)	0 (0)	>0.05	N/A
Constipation	3 (15.0)	0 (0)	5 (23.8)	1 (4.8)	>0.05	N/A
Alopecia	9 (45.0)	1 (5.0)	11 (52.4)	1 (4.8)	>0.05	>0.05
Neutropenia	9 (45.0)	2 (10.0)	11 (52.4)	1 (4.8)	>0.05	>0.05
Anemia	2 (10.0)	0 (0)	3 (14.3)	0 (0)	>0.05	>0.05

Quality of life (QoL) of patients were also assessed between the two groups at baseline and there was no significant difference at the last follow‐up.

## Discussion

Malignant pleural effusion is a common complication which occurs in approximately 15% of lung cancer patients and is a significant problem detrimental to a patient‘s quality of life.^6^ Intrapleural therapy by insertion of a catheter intercostally, and infusion of chemotherapeutic agents have been widely used for the treatment of symptomatic MPE.^13^ VEGF is a potent growth factor for endothelial cells and prompts the formation of new blood vessels. Cancer cells invade the pleura, produce large amounts of VEGF, and accelerate vascular permeability which play an essential role in malignant effusion formation.^6^, ^14^, ^15^ Consistent with this, the level of VEGF may be highly correlated to the formation and treatment results of MPE. Thoracocentesis by placing a catheter intercostally under appropriate local anesthetic is a commonly applied treatment option. This procedure is simple, safe and immediately relieves symptoms. Cytotoxic drugs such as cisplatin or nedaplatin are commonly infused intrapleurally for controlling MPE, but only 50%–60% patients respond to this treatment.^16^

Bevacizumab is a vascular endothelial growth factor A (VEGFA) monoclonal antibody which attenuates VEGFA dependent tumor blood vessels formation and inhibits tumor angiogenesis and has been used for the treatment of MPE. It can be administered by intravenous infusion or intrapleural infusion, but its optimal use has not yet been defined.^1^, ^17^, ^18^, ^19^ Serum VEGF level changes may correlate to bevacizumab treatment efficiency. In this study, intrapleural use of bevacizumab decreased serum VEGF levels and had a higher ORR than the intravenous method. In patients with DoR ≥ 3 months, their serum VEGF levels significantly decreased compared to baseline, and maintained lower levels.

The intrapleural group had a higher CR, DCR and longer DoR than the intravenous group, but there was no significance difference between the two groups. The reason for this may be that the size of the study was too small and more patients were required to be enrolled into the study. Bevacizumab‐induced toxicities including hypertension, proteinuria and epistaxis were higher in the intravenous group. This may have been because intrapleural infusion patients had a higher bevacizumab concentration in the thoracic cavity and lower blood concentration than intravenous infusion patients; and a relatively low blood bevacizumab level resulted in its low adverse effects.^20^

In summary, intrapleural infusion of bevacizumab had a higher ORR, longer DoR and less toxicity than intravenous infusion. The decreased level of serum VEGF 72 hours after bevacizumab treatment was closely related to the response rate and duration of response of pleural effusion. Intrapleural infusion of bevacizumab was safe and easily administered but further in depth studies are needed to explore its mechanism and efficiency.

## Disclosure

All authors declared there was no conflict of interest.
